# Supplementary material for: Content-rich biological network constructed by mining PubMed abstracts
Source: BMC Bioinformatics. 2004 Oct 8;5:147. doi: 10.1186/1471-2105-5-147 (PMC528731; doi:10.1186/1471-2105-5-147)
Supplement: Additional File 2 — The original results of the above study (non-essential files are deleted to keep the file size under the limit set by BMC bioinformatics). [file 1471-2105-5-147-S2.bz2 › chilibotAdditionalFile2/dip30/666ID10428814E11060/html/TNFRSF5_TRAF2.html]

 


 **TNFRSF5** and **TRAF2** 
  
Found 102 abstracts in PubMed, retrieved 30.  
 

 What does Google say? 
 PDF only 
| .edu only 

---

**Interactive relationship** (e.g. stimulation, inhibition, etc)

**Inhibitory relationship**- Previously, we found that a dominant negative  **TRAF2**  molecule inhibits CD40  [ **TNFRSF5** ]  mediated Ab secretion by the mouse B cell line CH12.LX.  Ref: 11907088 J Immunol, 2002
- Transiently expressed soluble trimeric CD40  [ **TNFRSF5** ]  cytoplasmic domain was isolated as complexes that contained  **TRAF2** , TRAF3, TRAF5, TRAF6, and the inhibitor of apoptosis protein c IAP1.  Ref: 11562359 J Biol Chem, 2001
- Consistent with this observation, catalytically inactive Tpl 2 also blocked CD40  [ **TNFRSF5** ]  mediated NF kappaB activation, which largely depends on  **TRAF2** .  Ref: 11932422 J Virol, 2002
**Neutral relationship**- we demonstrate that a fusion of LMP1 with CD40  [ **TNFRSF5** ]  recruits  **TRAF2**  intracellularly.  Ref: 12805217 EMBO J, 2003
- **TRAF2**  is used by CD40  [ **TNFRSF5** ]  and CD120b in distinct ways.  Ref: 11907088 J Immunol, 2002
- Cutting edge molecular mechanisms of synergy between CD40  [ **TNFRSF5** ]  and the B cell antigen receptor role for TNF receptor associated factor 2  [ **TRAF2** ]  in receptor interaction.  Ref: 12133933 J Immunol, 2002
- Furthermore, although both CD40  [ **TNFRSF5** ]  and CD120b can bind  **TRAF2** ,  **TRAF2**  dependent CD40  [ **TNFRSF5** ]  signals cannot substitute for  **TRAF2**  dependent CD120b signals in the activation of IgM secretion.  Ref: 11907088 J Immunol, 2002
- Upon receptor engagement,  **TRAF2**  is recruited to CD40  [ **TNFRSF5** ]  and translocates to lipid rafts in a RING finger dependent process, which enables the activation of downstream signaling cascades including c Jun NH 2 terminal kinase JNK and nuclear factor NF kappaB.  Ref: 12370254 J Exp Med, 2002
- Signaling via the TNF R family member CD40  [ **TNFRSF5** ]  has recently been shown to result in recruitment of  **TRAF2**  to plasma membrane detergent resistant microdomains lipid rafts as well as to subsequently initiate  **TRAF2**  degradation.  Ref: 11909853 J Biol Chem, 2002
- Both signals were partially dependent upon induction of TNF alpha and IL 6, and the cytoplasmic adaptor molecule TNF receptor associated factor 2  [ **TRAF2** ]  is involved in both R 848 and CD40  [ **TNFRSF5** ]  mediated differentiation.  Ref: 11067909 J Immunol, 2000
- The data presented here show that the shared binding site for  **TRAF2**  and TRAF3 accounts for receptor internalization, and the majority of signaling through CD40  [ **TNFRSF5** ] , but is redundant with the TRAF6 binding site for activation of p38 and NFkappaB signaling pathways.  Ref: 11754002 Eur J Immunol, 2002
- The critical role of TRAF1 in the regulation of  **TRAF2**  dependent JNK signaling is particular to the TRAF binding domain of LMP1, since a homologous region in the cytoplasmic tail of CD40  [ **TNFRSF5** ]  or the TRADD interacting domain of LMP1 signal on the JNK axis independently of TRAF1 status.  Ref: 12502848 J Virol, 2003
- These experiments revealed that anti CD40  [ **TNFRSF5** ]  stimulation of NF kappaB and IL 6 gene transcription requires specific amino acid residues in the cytoplasmic region of CD40  [ **TNFRSF5** ]  involved in the recruitment of  **TRAF2** .  Ref: 11886848 J Biol Chem, 2002

**Non-interactive relationship** (e.g. studied together, co-existance, homology, etc.)

- Comparison of the interactions of CD40  [ **TNFRSF5** ]  with TRAF3 vs.  **TRAF2**  suggests that CD40  [ **TNFRSF5** ]  may assume different conformations when bound to different TRAF family members.  Ref: 10984535 Proc Natl Acad Sci U S A, 2000
- To define the role of TRAF proteins in CD40  [ **TNFRSF5** ]  dependent isotype switching in B cells, we introduced wild type WT and mutant CD40  [ **TNFRSF5** ]  transgenes that lacked the binding motifs for TRAF6 CD40deltaTRAF6,  **TRAF2**  and TRAF3 CD40deltaTRAF2 3, or both CD40deltaTRAFs into B cells of CD40  [ **TNFRSF5** ]  mice.  Ref: 12354380 Immunity, 2002
- In this study, using isothermal titration calorimetry ITC, we report thermodynamic characterization of the interaction between  **TRAF2**  and monomeric peptide sequences from TNFR members, including TNFR2, CD40  [ **TNFRSF5** ] , CD30, Ox40, and 4 1BB, and the Epstein Barr virus EBV transforming protein, latent infection membrane protein 1 LMP1.  Ref: 10908665 Proc Natl Acad Sci U S A, 2000
- However, disruption of the  **TRAF2**  binding site in the cytoplasmic domain of CD40  [ **TNFRSF5** ]  does not diminish the ability of CD40  [ **TNFRSF5** ]  to stimulate Ab secretion, nor is this mutation able to circumvent the inhibition of Ab secretion by dominant negative  **TRAF2** .  Ref: 11907088 J Immunol, 2002
- Role of TNF receptor associated factor 2  [ **TRAF2** ]  in the activation of IgM secretion by CD40  [ **TNFRSF5** ]  and CD120b.  Ref: 11907088 J Immunol, 2002
- We show here that CD40  [ **TNFRSF5** ]  mediated  **TRAF2**  degradation requires the zinc binding RING domain of  **TRAF2**  and is preceded by  **TRAF2**  ubiquitination, suggesting that the  **TRAF2**  RING may promote ubiquitination although the RING itself is not a target of ubiquitination.  Ref: 11909853 J Biol Chem, 2002
- These results suggest that binding to  **TRAF2**  and or TRAF3 but not TRAF6 is essential for CD40  [ **TNFRSF5** ]  isotype switching and activation in B cells.  Ref: 12354380 Immunity, 2002
- These actions were abrogated by the CD40L blocking antibody TRAP  [ **TRAF2** ]  1 and the CD40  [ **TNFRSF5** ]  blocking antibodies M2 and M3, showing that activation was mediated by CD40L binding to platelet CD40  [ **TNFRSF5** ] .  Ref: 12676820 Circ Res, 2003
- Several approaches show that ubiquitination and proteasomal activity are integral to  **TRAF2**  degradation, and inhibition of this process potentiates CD40  [ **TNFRSF5** ]  signaling.  Ref: 11909853 J Biol Chem, 2002
- Stable transfectants expressing wild type or mutant forms of the cytoplasmic domain of CD40  [ **TNFRSF5** ]  suggested that TRAF3, but not  **TRAF2** , binding was essential for CD40  [ **TNFRSF5** ]  mediated RANTES expression.  Ref: 12122011 J Biol Chem, 2002
- The binding site for  **TRAF2**  and TRAF3 but not for TRAF6 is essential for CD40  [ **TNFRSF5** ]  mediated immunoglobulin class switching.  Ref: 12354380 Immunity, 2002
- Although TRAF1 can displace  **TRAF2**  and CD40  [ **TNFRSF5** ]  from raft fractions, it promotes the ability of  **TRAF2**  activate signaling over a sustained period of time.  Ref: 12370254 J Exp Med, 2002
